# Biocompatible and Biodegradable Polymer Optical Fiber for Biomedical Application: A Review

**DOI:** 10.3390/bios11120472

**Published:** 2021-11-23

**Authors:** Yue Wang, Yu Huang, Hongyi Bai, Guoqing Wang, Xuehao Hu, Santosh Kumar, Rui Min

**Affiliations:** 1 Center for Cognition and Neuroergonomics, State Key Laboratory of Cognitive Neuroscience and Learning, Beijing Normal University at Zhuhai, Zhuhai 519087, China; 201911039713@mail.bnu.edu.cn (Y.W.); 201911039085@mail.bnu.edu.cn (Y.H.); 2 College of Electronic Engineering, Heilongjiang University, Harbin 150080, China; baihongyi08@hlju.edu.cn; 3 College of Microelectronics, Shenzhen Institute of Information Technology, Shenzhen 518172, China; wanggq@sziit.edu.cn; 4 Research Center for Advanced Optics and Photoelectronics, Department of Physics, College of Science, Shantou University, Shantou 515063, China; xhhu3@stu.edu.cn; 5 Shandong Key Laboratory of Optical Communication Science and Technology, School of Physics Science and Information Technology, Liaocheng University, Liaocheng 252059, China; santosh@lcu.edu.cn

**Keywords:** biocompatible, biodegradable, polymer optical fiber, biomedical application

## Abstract

This article discusses recent advances in biocompatible and biodegradable polymer optical fiber (POF) for medical applications. First, the POF material and its optical properties are summarized. Then, several common optical fiber fabrication methods are thoroughly discussed. Following that, clinical applications of biocompatible and biodegradable POFs are discussed, including optogenetics, biosensing, drug delivery, and neural recording. Following that, biomedical applications expanded the specific functionalization of the material or fiber design. Different research or clinical applications necessitate the use of different equipment to achieve the desired results. Finally, the difficulty of implanting flexible fiber varies with its flexibility. We present our article in a clear and logical manner that will be useful to researchers seeking a broad perspective on the proposed topic. Overall, the content provides a comprehensive overview of biocompatible and biodegradable POFs, including previous breakthroughs, as well as recent advancements. Biodegradable optical fibers have numerous applications, opening up new avenues in biomedicine.

## 1. Introduction

Since the 1990s, optical fiber systems have been widely used in data transmission due to advancements in laser, optical fiber amplifier, and optical fiber technology [1]. With advantages such as no radiation, immunity to electromagnetic interference (EMI), and ease of multiplexing [2], optical-fiber-based technology is becoming more prevalent in a variety of sectors of our lives, including communication [3,4], microwave generation [5,6], mechanical inspection [6,7], and earthquake early warning [7,8]. Silica optical fiber is the backbone of the global Internet, with wavelengths optimized between 1260 and 1650 nm and typically employing single-mode fiber (SMF) with a core diameter of 4~8 μm [9]. Only one mode propagates in SMF, resulting in a faster transmission speed and greater distance than multimode silica optical fiber. The core diameter of a multimode silica optical fiber is typically 50 or 62.5 μm, which enables the propagation of multiple light modes within the optimized communication window of 850 nm and 1300 nm [10,11]. Silica optical fiber transmission ranges can be customized by adding impurities, such as doping materials. Additionally, there are various diameters of silica multimode optical fiber for use in specialized sensing applications [12,13]. Due to the numerous advantages, conventional silica optical fibers are widely used in sensors [12,14,15,16], high-energy military weapons [17], high-speed internet communications, and cloud-based data services [18,19,20]. While silica optical fibers have gained significant attention and value in medical applications, such as endoscopes [21], optical coherence tomography (OCT), and heart rate monitors [22], they are intrinsically stiff and, therefore, cannot provide the required flexibility and biocompatibility for biomedical applications. Due to their fragility and susceptibility to bending or distortion, the high risk of fracture may adversely impact the user’s safety when implanted in tissues or attached to the skin. Additionally, once a silica optical fiber is implanted beneath the skin, the incompatibility of its mechanical and chemical properties results in blood–material interface adhesion, foreign-body and infectious reaction, chronic inflammation, and tissue damage [23].

Polymer optical fibers (POFs) are optical fibers made of polymer optical materials throughout. For short-range visible light transmission, conventional polymer optical fibers are made of PMMA with core diameters of 980 μm or 735 μm [24,25]. A PMMA POF has an optical attenuation of 0.15 dB/m near 650 nm, which is three orders of magnitude greater than that of a standard silica optical fiber (0.2 dB/km at 1550 nm). However, because POF has a larger numerical aperture (NA) than standard telecommunications-grade silica optical fiber, they are more convenient to connect and install, making them attractive for short-distance connectivity applications, such as fiber-to-the-home (FTTH) and healthcare systems [26]. The first and most widely used polymer optical fiber communication medium was PMMA. With the advancement of optical fiber technology, AGC (formerly known as Asahi Glass Co.) now retails several varieties of CYTOP-based graded-index polymer optical fiber (GI-POF) under the trade name “Fontex”. This type of optical fiber has a double cladding structure and exhibits low attenuation and scattering losses due to the absence of the CH bond and a low refractive index. Unlike the POFs mentioned previously, the small-dimensional “Fontex” is transparent between 650 and 1300 nm and exhibits virtually minimal transmission loss. Additionally, it is more resistant to bending than conventional silica fiber, allowing for optical transmission through knots on fiber without disconnection [20]. Thus, “Fontex” is a type of optical fiber that is both reliable and safe for high-speed and large-volume data transmission. Additionally, polymer optical fibers are advantageous for sensing applications. For instance, researchers have discovered POFs’ extraordinary sensing capability, which includes pressure, humidity, temperature, and vibration [27,28,29].

Apart from the well-known applications mentioned previously, optical fibers have been used in medicine for decades, providing a safer and more efficient method of disease diagnosis, health monitoring [30], and clinical treatment [31,32]. With the development of advanced medical instruments in recent years, some optical fibers have found commercial applications in medical applications, and growing research is focusing on this promising technique. Compared with the conventional silica optical fiber, POFs possess many other advantages, such as lower Young’s modulus (~3.2 GPa for PMMA), higher failure strain, and better flexibility. The small size, low cost, nontoxicity, and electromagnetic insensitivity of biocompatible POFs make them ideal for devices applied in or near the body that shed some light on novel therapies in immunology, cardiology, neurology, oncology, and gastroenterology, among other fields [33,34,35]. For example, it is reported that wearable optical fiber based on fiber Bragg gratings can be used to monitor heart rate [36] and detect basic activities, such as walking, sitting, and squatting [15], which is promising for chronic disease prevention. Due to the increasing prevalence of diseases such as cancer, lithiasis, and angiocardiopathy, as well as the growth of minimally invasive surgery, there is an urgent need for more biocompatible, more photoconductive, and less invasive optical fibers, as well as nontoxic, tissue-like materials and low-cost, high-productivity, and versatile fabrication methods. In recent years, one has seen a surge in the use of polymer optical fibers due to their high elastic strain limit, flexibility, ease of manipulation, and low cost.

It is worth noting that a portion of biocompatible polymers are biodegradable, meaning they can be hydrolyzed or degraded into small molecules in a physiological environment [37,38]. As a result, it can further minimize damage to the host tissues, as additional removal surgery is not required. Natural materials are the best candidates for biocompatible and biodegradable optical fibers due to their superior optical and mechanical properties, nontoxicity, and intrinsic biodegradability that is proportional to the number of different components. Biocompatible optical fibers have been developed using protein, agarose, silk and spider fiber, cellulose, and cells [2,39,40,41]. In that, the bacteria-cell-based optical fiber exhibits the best biocompatibility and biodegradability due to its tissue-like nature. Synthetic polymers, such as poly (ethylene glycol) (PEG), poly (glycolic acid) (PGA), aliphatic polyesters of poly (lactic acid) (PLA), poly (lactic-co-glycolic acid) (PLGA), and poly (L-lactic acid) (PLLA), have been approved by the U.S. Food and Drug Administration (FDA) for medical applications, such as biosensing, drug delivery, and tissue engineering [42,43,44,45,46,47]. Along with low light attenuation and excellent transparency, this type of optical fiber has a low Young’s modulus and a high degree of flexibility, which allows it to be less cytotoxic and invasive to surrounding tissues. Hydrogel-based optical fibers are a research focus due to their porous structure and high water content that is compatible with the extracellular matrix [48]. This type of optical fiber is reported to have an excellent tensile strength of up to 2.27 Mpa, the ability to stretch more than nine times its original length, and the ability to self-heal [49]. Additionally, hydrogel optical fibers can remain functional for a controllable period of time in a physiological fluid environment after being implanted into free-moving mice [50]. Hydrogels perform poorly in comparison to polyesters and other thermoplastics in terms of optical properties and fabrication operability [38]. However, coating hydrogels on thermoplastic polymer fibers appears to be an ideal solution for tissue damage mitigation, as it softens the surface of the fibers and mimics the water content of the tissue. The advancement of medicine, particularly neuroscience, has increased the demand for multifunctional optical fibers, as the activities of cells or organs are influenced by the co-ordination of multiple signals, such as chemical signals, electrical signals, and neurotransmitters [51,52]. Multifunctional optical fibers allow for the integration of waveguides, microfluidic channels, and electrodes, enabling simultaneous interrogation of chemical, mechanical, and electrical signals without significant invasion. More precisely, they are capable of deep tissue delivery of drugs, nutrients, and viral vectors via hollow channels [53,54].

Many articles and reviews have been written about biocompatible and biodegradable optical fibers, that have become a hot research topic and are rapidly developing in recent years. In 2018, Nazempour, R. et al. published an overview on the development of bio-compatible and implantable optical fibers and waveguides for biomedicine, which included a good number of examples and illustrations, clear organization, and expression, as well as informative discussion and outlooks [55]. Gierej, A. et al. presented a systematic review of fabrication processes and discussed issues that may influence them, such as biomaterial properties and other considerations [56].

**Figure 1:**
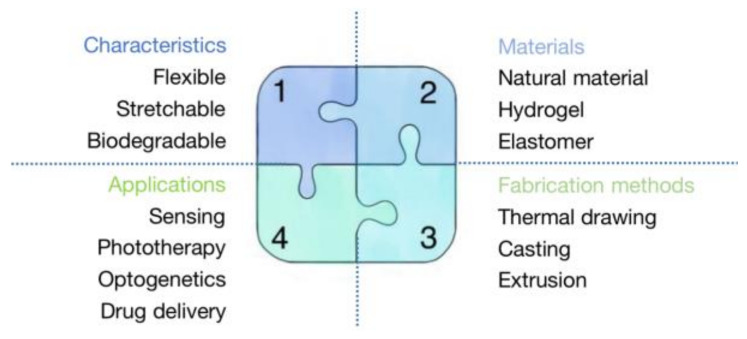
A summary of this review about biocompatible and biodegradable polymer optical fibers.

In this review, we focus on the differences between optical fiber and waveguide that have been overlooked in the majority of previous reviews. In addition, to keep this article focused and well-organized, we only use biocompatible and biodegradable optical fibers made of polymers. It is worth noting that we treated biomaterials, optical fiber fabrication methods, and their biomedical applications equally in order to make our review informative and appropriate for anyone seeking a thorough understanding of the topic. We present our article in an instructive and logical manner that is useful for researchers who want a broad perspective on the subject and also for readers who do not have a thorough understanding of the topic. We also summarize a number of informative tables and conclusive diagrams to enrich the article. Finally, the content provides a broad overview of biocompatible and biodegradable POFs, including earlier breakthroughs, as well as recent advancements. Section 2 divides constituent materials into five categories: natural materials, hydrogels, synthetic materials, elastomers, and multi-materials. We summarize the optical and mechanical properties, advantages, and limitations of each material using a variety of common and advantageous optical fibers as examples. Because some chemicals appear visually similar, we provide skeletal formulas in addition to their physical and chemical properties to highlight the differences between them. Section 3 focuses on the most commonly used biocompatible and biodegradable optical fiber fabrication methods, such as thermal drawing, casting, extrusion, and other approaches derived from extrusion. In this section, we will go over the fabrication steps, precautions, benefits, and drawbacks of each fabrication method in detail. Section 4 summarizes the critical applications of biocompatible and biodegradable optical fibers in medicine, such as sensing, phototherapy, neural recording, drug delivery, and optogenetics. In the conclusion, we clearly and concisely summarize the benefits and drawbacks of biocompatible and biodegradable POF. Although biocompatible and biodegradable optical fibers perform worse than silica optical fibers in terms of light-guiding efficiency, and there are challenges in complex structure fabrication methods and minimally invasive implanting surgery, biocompatible and biodegradable optical fibers have a bright future in biomedicine. Figure 1 shows a summary of this review about biocompatible and biodegradable polymer optical fibers.

## 2. The Material of Polymer Optical Fiber

Biocompatible and biodegradable optical fiber can be made from a variety of materials. Despite the fact that standard fused silica glass is the most commonly used material in current fiber manufacturing, its lack of biocompatibility and biodegradability is an enormous challenge in many biomedical applications. In recent years, many novel polymer optical fiber materials have been developed. We classify polymer materials into five categories and illustrate the corresponding representative materials in the following statement (as Table 1 shown). Natural materials [57,58,59,60], hydrogel [61,62], synthetic [44,45,63,64,65], elastomers [66,67], and multifunctional [53] optical fibers not only have greater biocompatibility and biodegradability, but also have unique properties that are suitable for various biomedical applications.

### 2.1. Natural Materials

Natural biomaterials, such as proteins, polysaccharides, and cell materials, have been used in a variety of biomedical applications, not only for their superior performance characteristics, such as biocompatibility and nontoxicity, but also for their ease of processing. Additionally, certain natural fiber materials are used in biomedical fields because of their superior optical properties, including high transmittance and optical conductivity [45,68].

#### 2.1.1. Silk

Silk, which is derived from spiders or silkworms, is already a widely used material in clinical applications, such as wound healing, sutures, tissue engineering, and fabrics [57], owing to its biocompatibility and biodegradability. At the moment, reliable clinical research evidence indicates that, when impurities are completely removed, Bombyx mori silk fibroin will exhibit excellent biocompatibility [57]. Additionally, its high transparency, great flexibility, low light loss, and adjustable mechanical properties make it an ideal optical fiber material. In one report, the optical properties of silk fibroin extracted from silkworms were examined. The optical silk planar waveguides demonstrated a low optical loss of 0.22 dB/cm (632.8 nm) and a refractive index of 1.54–1.55 (632.8 nm) [69]. Furthermore, the mechanical properties of silk optical fiber can be adjusted to meet specific requirements by adjusting the proportions of various elements [70,71], and the silk optical fiber can be functionalized with bioactive molecules [72].

Spider silk protein is also an ideal optical fiber material [73]. Using genetic engineering technology, researchers have been able to express spider protein through bacteria [74,75], and optical fibers made of spider protein have also emerged. Xin et al. published an optical waveguide made of genetically engineered spider silk protein in 2017. Not only did they overcome the limitation of low production of natural spider silk, but also produced optical fibers with excellent optical and biocompatible properties. The optical loss of the spider silk protein optical fiber in mouse muscle can be as low as 1.9 ± 0.3 dB/cm. They also proved that this optical fiber had a low cytotoxicity in vitro and weak inflammatory response in vivo [76]. These examples show the potential application value of spider silk proteins in biocompatible and biodegradable materials.

#### 2.1.2. Cellulose

The natural material cellulose, which is abundant in plants, has numerous properties similar to silk and has been used in the fabrication of optical fibers. Several previous studies have demonstrated that cellulose has significant potential for drug delivery [58], chemical sensing (including novel nitrite (NO_2_) sensing [59] and gaseous ammonia sensing [60]), and electrochemical sensing.

Cellulose-based optical fibers exhibit high ion permeability and excellent light transmittance across a broad spectrum of light [60]. It is worth noting that the more cellulose in a material, the higher its modulus and tensile strength [40]. Thus, the cellulose content of these optical fibers can be adjusted to alter their mechanical properties. As is the case with other natural fibers, this type of fiber is biocompatible and biodegradable. Additionally, cellulose-based microstructural fibers can perform multiple functions, offering significant potential for use in biomedicine [40,77].

#### 2.1.3. Bacteria-Cell-Based

Along with conventional optical fiber, bacterial cell-based optical fiber has become available in recent years. An organism’s ideal biomaterial is a component of the organism itself. Bacteria-cell-based biomaterials are more biocompatible than conventional materials because they can be degraded and absorbed in a biophysical environment. Due to the fact that light interacts with biological cells, living bacteria cells are used to exploit biophotonic components that enable cells to function as optical detectors and test samples for sensing simultaneously [78]. It has been reported that bacteria-cell-based fibers exhibit excellent light propagation properties [41].

### 2.2. Hydrogel

Hydrogels, a type of hydrophilic polymer network, have attracted considerable interest in biomaterial research for many years [79] due to their superior properties, such as biocompatibility and versatility, and especially hydrophilicity. They can absorb water and expand to tens to thousands of times their dry weight, depending on the concentrations, molecular weight, and controllable ionic strength [80]. Additionally, they can be chemically stable or biodegradable, depending on the pH value, molecular interactions, composition ratios, and physical structure, among other factors [80,81,82]. Since the first hydrogels were successfully crosslinked in 1960 [83], hydrogels have been discovered to have applications in drug and cell carriers, wound dressing, tissue engineering, and emerging phototherapies. This section will discuss the materials used to make biocompatible hydrogel optical fibers and their associated properties.

Due to the high flexibility and stretchability of hydrogel optical fibers, they are well suited for use in wearable devices [84]. Additionally, due to their small size, high water content, low immunogenicity, and low toxicity [46,85], they are ideal for medical applications that require implanting into the body without causing significant damage to the host tissues, even in vivo [86]. Researchers have developed hydrogel optical fibers with increased flexibility, lower propagation loss, a longer function period, and a more controllable degradation rate [50,85,87]. These fibers can be used widely as drug and cell carriers [88], glucose and blood oxygenation level sensors [46,85,89], and light delivery, introducing optogenetics therapies, minimally invasive surgery, and photomedicine [45,46,62,90] one step closer to clinical practice.

#### 2.2.1. PEG Hydrogel

Polyethylene glycol, also known as polyethylene (PEO), is a widely used amphiphilic biomaterial due to its high water capacity (it readily absorbs up to 95% of its weight in water), similarity to the extracellular matrix, and low toxicity [91]. Additionally, due to PEG’s unique antifouling properties, it can inhibit the growth of skin cells and protein aggregates, making it an attractive candidate for biomedical applications [92]. Indeed, PEG and PEO are both ethylene oxide macromolecules with a different molecular weight, with PEG having the ideal molecular weight for biomedical applications (less than 20,000 g/mol). Due to their good properties of light-guiding efficiency (optical propagation loss of less than 1 dB/cm in mice [61]), optical transparency, mechanical flexibility, and ease of integration, PEG-based optical fibers are promising for biosensing and light-induced therapies.

When combined with other materials and fabrication techniques, PEG-based optical fibers provide a highly effective and minimally invasive method of continuous healthcare monitoring. The researchers developed an implantable glucose detector based on fibers and functionalized with glucose-sensitive motifs, such as phenylboronic acid (PBA), diboronic acid, and anthracene acid [93], and 3-(acrylamido)-phenyboronic acid (3-APBA) [46]. In theory, fiber probes equipped with functionalized aptamers and structures are capable of detecting a broader range of biomolecules, such as DNA, hormones, and proteins. For example, it has been reported that a taper PEG-based hydrogel fiber probe combined with silver nanoparticles (AgNPs) can be used to achieve higher-sensitivity dopamine monitoring on a larger scale [94].

#### 2.2.2. PEGDA Hydrogel

**Figure 2:**
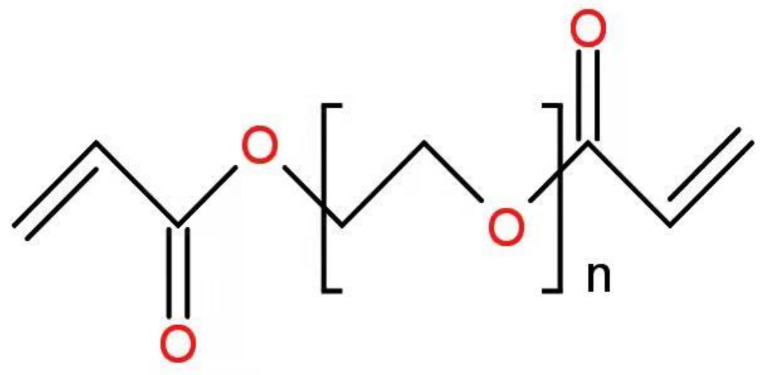
Chemical formula of PEGDA.

Optical fiber performance and application are limited by a lack of biocompatible materials and the shortcomings of existing fabrication techniques. Researchers have overcome these limitations in recent years by developing glucose-monitoring fibers with high sensitivity, high glucose selectivity, low cost, and rapid response time [46,95]. To illustrate, one type of optical fiber probe based on PEGDA (*n* = 1.351) is primarily composed of PEGDA, 3-APBA, acrylamide probe (AM), and sodium phosphate dibasic. PEGDA’s chemical formula is shown in Figure 2.

#### 2.2.3. PAAm Hydrogel

Due to its excellent properties, such as high stretchability, low light propagation loss, low modulus, and long lifespan, optical fibers based on poly(acrylamide)–alginate (PAAm–Alg) hydrogel are an effective method of strain sensing [62]. As a result, this highly functionalized optical fiber is well suited for optogenetics. Table 2 summarizes the names and main properties of the mentioned hydrogel materials.

### 2.3. Synthetic Polymers

Various synthetic biocompatible polymer materials have been developed and introduced over the last several decades due to their unique properties, which include controllable degradation profiles, low propagation loss, and ideal mechanical properties. Synthetic polymers can be degraded and absorbed or cleaned in the biophysical aqueous environment, with little or no toxicity remaining in the body. Thus, it is ideal for use in implantable medical devices, as it eliminates the need for additional surgery to remove the implant. Table 3 shows the main characteristics of the synthetic polymer materials.

#### 2.3.1. PLA, PGA, PLGA

PGA polymers, aliphatic polyesters of PLA polymers, and their copolymer PLGA are all capable of delivering light into deep tissues. (The refractive index of the majority of soft tissues ranges between 1.33 and 1.51 [63,64].) Hydrolytic and zymolytic processes in a biophysical environment can completely degrade PGA, PLA, and PLGA polymers into mini-toxic or nontoxic small molecules that can then be absorbed or removed. Casting, drawing, press molding, or 3D printing are used to fabricate optical fibers [44,45,65]. They are designable and versatile because of the degradation rate (which can range from a few minutes to more than a year [103]). The biofunctionalities, mechanical, and optical properties can all be adjusted by adjusting the molecular weight, enantiomeric forms, lactide/glycolide unit ratios, or the additional moieties or end groups [47]. They have been widely used as injectants and implants to deliver drugs and regenerate tissue.

For instance, a PLA-based planar optical fiber can act as a waveguide for photochemical tissue bonding treatment in deep tissues. In a previous experiment, a PLA-based optical fiber delivered 7.5% of the input light into bovine tissues and demonstrated the potential to increase the therapy depth in human skin from 1.3 mm to 2.5 mm [44]. Recently, researchers developed a new PLA-based optical waveguide for full-thickness (>10 mm) skin incisions, ten times the thickness of conventional surface illumination [65]. The proposed comb-shaped planar optical waveguide has a width of 650 μm and a thickness of 240 μm, with a loss coefficient of 0.16 dB/cm. To achieve illumination of deeper tissues, attenuation should be reduced further. There are several possible approaches: (1) increasing the optical fiber’s width; (2) increasing the optical fiber’s thickness; and (3) altering the sidewall patterns.

Additionally, researchers have created biodegradable and biocompatible optical fibers using extrusion printing from PLA and its copolymers, such as PLGA, and PLA with polycaprolactone (PCL). At 37 °C, the printed PLA and PLA-co-PCL optical fibers are malleable and amorphous. The propagation loss is 0.02–0.26 dB/cm in air and 0.14–0.73 dB/cm in tissue at 405–520 nm light, which is significantly less than that of other types of optical fibers (1.5–1.64 dB/cm in air at 473–532 nm light [44,65]). It has been demonstrated that it is capable of delivering 405 nm light into 3D cell cultures and performing photocleavage at a depth of over 8 cm. The diameter of the printed fiber is precisely controlled by the nozzle size, the ink viscosity (as determined by the material ratio), temperature, and pressure [104].

#### 2.3.2. PLLA

Optical fibers made of PLLA have been extensively used in tissue engineering and are expected to be implanted or injected into brains. Due to their optical transparency, ideal flexibility, and controllable biodegradability, these optical fibers shed light on neural biomedicine. A group of researchers conducted an experiment in which they implanted PLLA-based optical fibers into the brains of both in vitro and in vivo mice and evaluated their mechanical, optical, and biocompatible properties. The fabricated optical fiber with a diameter of 220 μm has a lower bending stiffness (1.5 × 10^4^ N/m) than conventional silica fiber (2.4 × 10^4^ N/m), which means it can cause less damage to nearby tissues. Although the attenuation of 473 nm light increased with time and fiber length, total internal reflection at the PLLA/brain interface can still ensure effective photon propagation within the fiber [45].

### 2.4. Elastomers

Hydrogel materials have a wide range of applications due to their excellent biocompatibility and flexibility. However, the biggest drawback is that they cannot work in a dry environment for long periods of time, whereas elastomer-based optical fibers do not have this limitation. Table 4 shows the main characteristics of the elastomer materials.

#### 2.4.1. PDMS Polymers

PDMS (polydimethylsiloxane) is an organic elastomer composed of silicone. Along with some fundamental properties, such as chemical inertness, good elasticity, and thermosetting [109], it possesses extraordinary optical properties. Prajzler et al. reported PMDS waveguides to have a low refractive index (*n* = 1.41, λ = 589 nm) [66] and low optical absorption loss (1.8 dB/cm, λ = 532 nm; 1.0 dB/cm, λ = 633 nm) [110]. The same group also reported POF splitters with silicon optical elastomer LS-6943 as core and PDMS as cladding, which have a core refractive index around 1.433 and cladding refractive index around 1.416 at 532 nm wavelength [111]. Shentu et al. reported a no-core PDMS fiber with strain sensitivity of 3.5070/ε (large-scale strain from 0% to 40%) [112]. Guo et al. reported a PDMS fiber with the ability to enlarge strains (100%) and high reproducibility (over 6000 cycles) [113].

Traditionally, PDMS was prepared using soft lithography, but this method has several drawbacks. Soft lithography is limited in its ability to create long gracile circular waveguides. Recently, an improved modified fiber-drawing approach was reported, which can overcome the aforementioned soft lithography constraint [78]. However, there are still issues that must be resolved before this material can be used more widely. With the exception of a few polymers, such as polydimethyl–diphenyl siloxane (PDM–DPS), the majority of PDMS polymers are incompatible with other polymers, causing difficulties during the drawing process. As a result, new technologies have been developed to enable the use of PDMS-based optical fibers in a broader range of applications.

#### 2.4.2. POC-POMC Optical Fiber

POC-POMC optical fibers are biodegradable because they are composed of a biodegradable poly(octamethylene maleate citrate) (POMC) core and a poly(octamethylene citrate) (POC) cladding layer, which can be degraded within a few weeks [16]. It demonstrates excellent light transmission properties in previous work, with a light propagation loss of as little as 0.4 dB/cm [67]. Additionally, POC and POMC exhibit excellent elasticity and softness in tensile tests, with initial moduli of 3.79 ± 0.45 and 4.35 ± 0.51 MPa, tensile strengths of 1.54 ± 0.16 and 1.78 ± 0.20 MPa, and elongations of 62.5 ± 5.3 % and 92.5 ± 10.2%, respectively [67].

### 2.5. Multifunctional Fibers

Researchers have been attempting to understand how our brains learn [114], memorize, and make decisions [115] when multiple signals, including electrophysical, chemical, and other signals, are comodulated. With the advancement of neuroscience, optogenetics [116], and photopharmacology [117], there is a growing need for multifunctional and biocompatible devices capable of recording multiple signals with high resolution and minimal invasion. Using biocompatible multifunctional fiber probes, researchers have recorded spinal cord [118] and brain circuits [53,54,119].

A group of researchers developed implantable multifunctional fiber probes for simultaneous optical, electrical, and chemical interrogation of neural circuits in free-moving mice that remain functional for two months following the operation [54]. The fiber probe was fabricated using a thermal drawing process via a combination of polycarbonate (PC, refractive index *n* = 1.586, glass transition temperature T_g_ = 150 °C [120]) and cyclic olefin copolymer (COC, *n* = 1.53, T_g_ = 158 °C [121]) as waveguide and recording electrode. The integrated fiber electrode for recording, waveguide for light transmission, and microfluidic channel for drug delivery in a single probe is proposed. The optical loss of this fiber at 473 nm was found to be 2.4–4 times less than that of previously reported polymer-based fibers [122]. Meanwhile, the electrodes’ impedance at 1 kHz was between 0.5 and 5 MΩ, which is also quite low. Additionally, the drug delivery efficiency was unaffected by the fiber probe being bent 90°. Another research group succeeded in fabricating miniaturized multifunctional fiber probes with a diameter of 180–220 μm and a higher density of electrodes, microfluid channels, and waveguides [53]. The reported conductive polyethylene with 5% graphite (gCPE, melting temperature ~123 °C) demonstrates superior conductivity (sheet resistance 0.8 kΩ for gCPE vs. 3.3 kΩ for CPE) that enables the size to be reduced.

A team of researchers recently developed an adaptive, multifunctional, and biocompatible hydrogel fiber probe for long-term optogenetic interrogation. It remained functional in freely moving mice six months after implantation. Due to the numerous mechanical and chemical advantages of the hydrogel-based hybrid probe, such as low bending stiffness and stability in a physiological environment, it is capable of minimizing foreign body responses and causing minimal damage to host tissues. Individual microscale fiber probes were fabricated using thermal drawing, integrating electrodes from tin microwires, microfluid channels from poly(etherimide) (PEI) tubes, and waveguides constructed from PC core and COC cladding. To achieve optimal mechanical and chemical compatibility with the surrounding neural tissues, a poly(acrylamide)–alginate (PAAm–Alg) hydrogel matrix was chosen to hybridize multiple microscale functional fibers into a multifunctional probe, due to its mechanical robustness (fracture toughness: 500 J/m^2^ [123]), tissue-like softness (shear modulus: 5.5 kPa), long-term chemical stability in the physiological environment, and robust interfacial integration with individual functional microfibers [119].

## 3. The Fabrication Method

The optical fiber’s properties are highly dependent on the fabrication process. With the advancement of technology, numerous fabrication methods have emerged, including thermal drawing, casting, extrusion, 3D printing, and spinning. Thermal drawing, a process that involves heating, drawing, and cooling a well-structured preform into extended fibers with microstructures, is a cost-effective and widely used method for fabricating optical fiber. Thermal drawing is widely used in optical fiber fabrication, particularly in the booming multifunctional fiber fabrication industry, due to its ability to integrate diverse materials and complex structures. The second part will introduce extrusion, extrusion-based 3D printing, and spinning, while also providing references and comparisons. Extrusion is a widely used fabrication process in which the prepared solution or melt is forced through the nozzle and forms the optical fiber in the air. In practice, 3D printing and spinning are both additive processes based on extrusion. As a result, we grouped them together to emphasize the connections between them. In comparison to the previous methods, casting is the simplest, as it involves the solution or melts forming the shape of the fiber in a specially designed mold. It is capable of fabricating optical fibers of any shape from a variety of materials, including hydrogel, elastomer, silk fibroin, and others. However, fabricating functional optical fibers with complex structures via casting remains challenging, impeding the application of this efficient and low-cost method.

This section will discuss the advantages and disadvantages of the front five fabrication methods: thermal drawing, extrusion, extrusion-based 3D printing, spinning, and casting.

### 3.1. Thermal Drawing

Thermal drawing has been widely used in fiber fabrication due to its numerous advantages, including high productivity, superior uniformity and scalability, and the ability to integrate a variety of materials and complex structures. In comparison to other fabrication methods, thermal drawing can generate thousands of fibers with a length of up to tens of kilometers in a single process [124].

Thermal drawing begins with the preparation of a preform, which is a larger version of the designed fiber. Indeed, the preform has a direct effect on the optical and mechanical properties, structure, and lifespan of the thermally drawn fiber. As a result, in order to obtain the desired fibers, it is necessary to develop a well-structured preform in which the appropriate variety and quantity of constituent materials are precisely located in the appropriate position. Preforms can be produced in a variety of ways, including molding [125], extrusion [126], 3D printing [127,128], and thin-film rolling [54].

When selecting materials for thermal drawing, several criteria should be considered. For example, the constituent materials should have similar glass transition temperatures (T_g_), melting temperatures (T_m_), and thermal expansion coefficients to ensure that the co-drawing process proceeds smoothly and successfully. In reality, the most critical factor to consider is the viscosity compatibility of the materials [129]. Additionally, the materials used for fiber cladding should be thermoplastic and have a high viscosity (10^4^–10^8^ Pa·s at drawing temperature) and molar mass (10^4^–10^5^ g/mol) [56] in order to maintain the preform’s complex architecture during the drawing process. Individual components can be manufactured in a variety of ways, depending on the physicochemical properties of the materials and the desired properties of the fabricated preform. Take the rapid-developing multifunctional fiber as an example; the preform consists of different types of materials, such as metal, polymers, semiconductors, and insulators. Thus, they require different machining procedures, which allow for appropriate slots to be rightly constructed and positioned within the polymer slabs. For instance, shaping semiconductors, such as silica, usually requires milling and powder consolidation due to their brittle mechanical properties. Alternatively, semiconductor thin films can be formed on the surface of polymer films by evaporation and rolling [129]. Casting is a widely used method of preform fabrication due to its simplicity and universality, which is also widely used in fiber fabrication [130]. Solid materials, such as metal, can be manufactured by powder consolidation supplemented by milling. Table 5 shows the fabrication methods of different types of preform constituents. Once all of the components have been prepared, the final step is to combine the separated slabs via thermal consolidation to obtain the desired preform.

**Figure 3:**
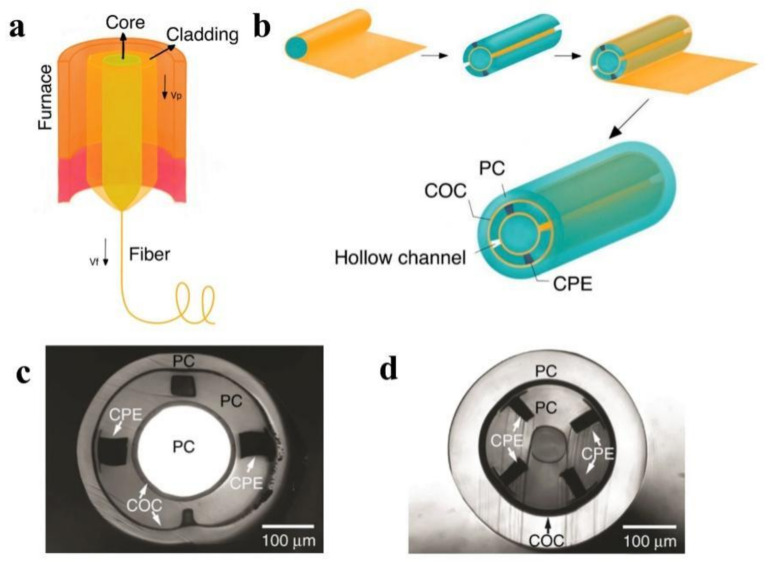
(**a**) A schematic of the thermal drawing process. (Adapted from ref. [132]). (**b**) A schematic of the preform fabrication process of design I. (Adapted from ref. [55]). (**c**) A cross-sectional optical image of design I, which has one cylindrical waveguide, two electrodes, and two microfluidic channels. (Reprinted with permission from ref. [40]). (**d**) A cross-sectional optical image of design II, which has one surrounding waveguide, four electrodes, and one microfluidic channel. (Reprinted with permission from ref. [40]).

Figure 3a illustrates the thermal drawing process. The procedure was divided into three distinct stages. To begin, the preform was suspended above the drawing tower’s tube furnace, and a weight may be attached to the preform’s end. Second, the furnace temperature was maintained 50–100 °C above the T_g_ of the cladding material for 30–60 min. The bottom of the preform softens and necks as time and temperature increase due to the pulling of the attached weight. Thirdly, the fiber was collected at a constant speed using a rotary capstan, while the preform was fed into the furnace at a much slower speed. The diameter of the obtained fiber is shown as below:

(1) $$D_{fiber}=D_{preform}\times \sqrt{\frac{v_{p}}{v_{f}}}$$

Here, *Dfiber* refers to the diameter of the drawn fiber, *Dpreform* refers to the diameter of the preform, *v_p_* refers to the feeding speed of the preform, and *v_f_* refers to the rotary speed. So, the diameter of the drawn optical fiber is controlled by the diameter of the preform, the feed rate, and the collection rate.

A preform can be drawn to produce fibers with diameters ranging from hundreds of micrometers to several millimeters. Additionally, it is worth noting that the internal core’s diameter can reach the nanoscale [131].

For example, one type of multimodality fiber probe based on PC-COC-CPE was fabricated via thermal drawing for optical, electrical, and chemical interrogation of neural circuits [54]. In the first step of design I, several COC sheets and PC sheets were rolled around a PC rod, annealed, and hardened. Then, four rectangular grooves were drilled on the cylinder, two of which will be filled with conductive CPE to serve as two electrodes and the remaining two will be hollow to accommodate microfluidic channels. Finally, the previous preform was wrapped with PC, COC, and additional PC sheets. In the case of design II, the optimized concentric structure contributes to the probe’s dimension reduction. As a result, the electrode density was increased. The preform was formed by rolling COC and PC sheets around a ceramic mandrel, machining four grooves with CPE, and then rolling COC and PC sheets around. The fabrication process is illustrated in Figure 3b. Both preforms were annealed at 190 °C and then thermally drawn at 240 °C.

**Figure 4:**
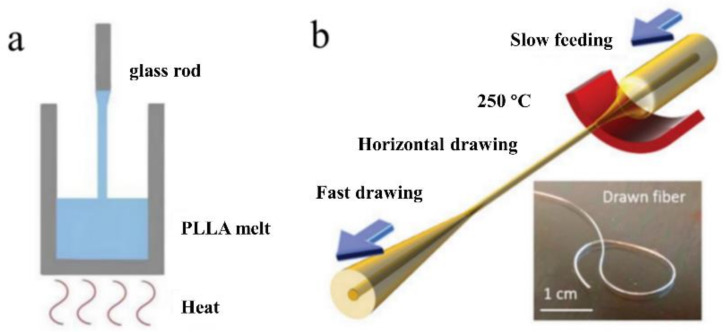
(**a**) A schematic of the simplified thermal drawing process of the PLLA-based optical fiber. (Adapted from ref. [45]). (**b**) A schematic of the horizontal thermal drawing process of TPE-based fiber. (Reprinted with permission from ref. [133]).

However, not all thermal drawing processes are identical to those mentioned previously. For example, simplified thermal drawing without the use of a drawing tower was used to fabricate biodegradable PLLA optical fibers for neural interfaces [45]. The liquid precursor was composed of melted crystalline PLLA polymer powders. As illustrated in Figure 4a, the precursor was later drawn into optical fibers and cooled using a glass rod. The drawing speed had a direct effect on the diameters of the drawn optical fibers. Additionally, not every drawing setup is vertical. As an illustration, the recently reported highly stretchable, small-diameter optical fibers based on thermoplastic elastomers (TPEs) were manufactured via horizontal thermal drawing [133]. One reason is that the preform has a small diameter (a few millimeters) and the setup simplifies the control of the drawing parameters. Another reason is that TPEs have a low viscosity, making it difficult to support the weight of the bottom while maintaining the preform’s structure. Figure 4b illustrates the drawing process schematically.

As mentioned previously, it is critical that the thermomechanical properties of constituent materials are compatible. It is, however, an impediment to the integration of low-impedance metallic electrodes and low-loss polymer waveguides. Historically, electrode materials have been limited to carbon-based polymer composites and drawable metals, such as tin [54], which have low conductivity and, thus, require a larger fiber probe. Recently, a group of researchers introduced two approaches based on thermal drawing to resolve this dilemma: iterative thermal drawing with low T_m_ indium and metal convergence drawing with previously undrawable high T_m_ tungsten [134]. The multifunctional fiber probes were capable of recording neural circuits in real-time in mice for several weeks.

### 3.2. Extrusion, Extrusion-Based 3D Printing, and Spinning

**Figure 5:**
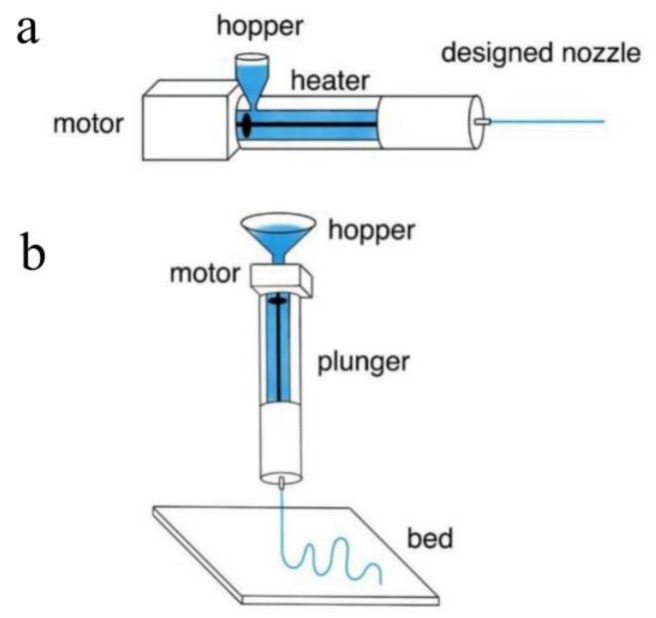
(**a**) A schematic showing the extrusion process. (Adapted with permission from ref. [56]). (**b**) A schematic showing the extrusion-based 3D printing. (Adapted with permission from ref. [56]).

For many years, extrusion and extrusion-based three-dimensional printing have been extensively used in the fabrication of functional fibers or preforms. Extrusion is a process in which the precursor material, typically melted materials or preblended solutions, is forced through a nozzle. It can produce filaments or unclad fibers in a continuous fashion. 3D printing, which is an extruded-based additive processing, enables the production of more complex optical fibers. To a large extent, the shape of the extruded preform or fiber is determined by the extrusion die used or the nozzle pattern. Thus, by using a die with a holey pattern, it is possible to fabricate fibers with desired hollow channels via 3D printing [135]. Additionally, 3D printing enables the coextrusion of multiple materials and the production of step-index fibers with microstructures [136]. Extrusion and extrusion-based 3D printing are shown in Figure 5.

In theory, spinning is like extrusion in that it utilizes imposed forces to extrude solutions or melts. Several types of spinning have been developed to date, including wet spinning [88,137,138], dry spinning [139,140,141], electrospinning [142,143,144], microfluidic spinning [145], and dynamic crosslinking spinning [146]. Below the following sentences, the first three approaches have been thoroughly reviewed.

Microfluidic and dynamic crosslinking spinning are both efficient methods for mass producing well-structured functional optical fibers. They are, however, unable to manufacture the widely used core-sheath optical fibers. Wet spinning has been widely used to fabricate POFs due to its numerous advantages, including efficiency, controllability, and, most importantly, the ability to produce large quantities of core-sheath hydrogel fibers with ideal optical and mechanical properties in a continuous manner [73,137,138,147].

**Figure 6:**
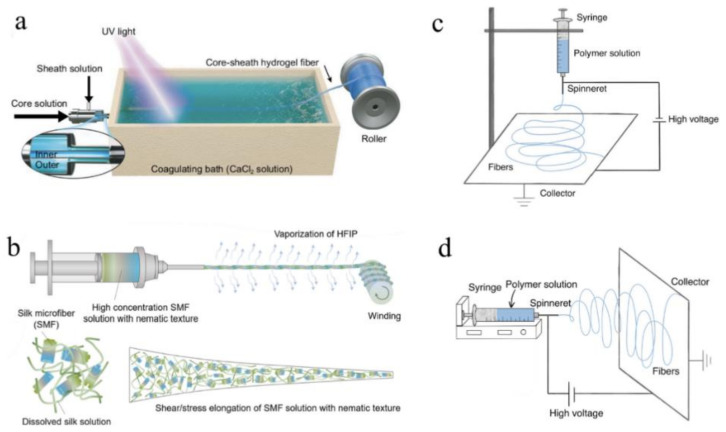
A schematic showing the (**a**) ILDAWS process. (Reprinted with permission from ref. [88]). (**b**) Bioinspired dry spinning process. (Reprinted with permission from ref. [140]). (**c**) Vertical electrospinning setup device. (Adapted from ref. [148]). (**d**) Horizontal electrospinning setup device. (Adapted from ref. [148]).

A group of researchers recently reported the fabrication of core-sheath optical fibers with exceptional properties using integrated light-triggered dynamic wet spinning (ILDWS) [88]. p(PEGDA-co-AAm) was chosen as the core material and Ca–alginate as the sheath material in this work based on optical transparency, refractive index, and solution viscosity. Figure 6a depicts the fabrication process. The procedure was comprised of three steps. To begin, the spinning solutions were prepared, including the core spinning solution, which contains dissolved PEGDA and AAm monomer in deionized water, and the sheath spinning solution, which contains aqueous Na–alginate. Second, the solutions were poured into a bath of CaCl_2_ solution to coagulate them. Ionic crosslinking was used to gel the sheath solution in the bath, while UV crosslinking was used to crosslink the core solution under 360 nm UV illumination. Finally, the core-sheath fibers were assembled into a cylinder. The monomer weight ratio and extrusion rate were used to control the optical properties of the fibers.

Dry spinning, another method of spinning, is capable of fabricating optical fibers in a single step. To summarize, the high vapour pressure solution is prepared and extruded into the air via a needle at a specific temperature. Following evaporation, the extruded solution dries and transforms into desired fibers. Mass transfer, heat transfer, and filament stress are all critical factors in the process [139,141].

For instance, bioinspired dry spinning was used to fabricate polymorphic regenerative silk fibers that retain the structural mechanical properties of natural materials [140]. The silkworm cocoon was boiled in 30 min changes of HaHCO_3_ solution, and then washed with distilled water during the fabrication process. After air drying at room temperature, the silk fibers were immersed in HFIP solution and kept at 60 °C for 7–15 days in airtight containers. Finally, the prepared solution was extruded into the air to produce the desired optical fiber. Figure 6b depicts the spinning process.

Electrospinning, another extrusion-based fabrication method for mass industrial production, has been widely adopted due to its ability to continuously produce a variety of fibers with diameters ranging from nanoscale to submicron, as well as its simplicity for nonfunctionalized fibers. This technique utilizes strong electrostatic forces to extrude solutions or melts in order to produce optical fibers with smaller diameters and greater surface areas. The solution viscosity, polymer concentration, voltage value, air humidity, work distance, and surface tension of polymer solutions or melts all have an effect on the fabrication process and the properties of the resulting optical fibers [149,150]. Thus, the fabrication process is typically carried out in a closed chamber with strict control over the temperature and humidity of the air. The method can be used to fabricate nanofibers from polymers, ceramics, metals, and composites [151]. For instance, a wide variety of polymers, such as PGA [142], PLGA [143], PLLA [144], poly (vinyl alcohol)/cellulose acetate (PVA/CA) [152], and silk [153,154], can be electrospun into optical fibers with ideal properties. Electrospun fibers are believed to have promising applications in biomedicine [155], including drug delivery, implants, and tissue engineering.

A spinneret, a syringe pump, a high DC voltage source (1–30 V in laboratory conditions), and a fiber collector system comprise an electrospinning system [151]. In brief, when a high voltage is applied to the polymer solution or melt, it emerges from a nozzle as electrically charged droplets. When electrostatic repulsion overcomes the surface tension of the charged droplet, the charged droplet stretches to the critical point of liquid stream eruption. The solution or melt is extruded at a controlled rate using the forces of a pump [156]. Between the syringe and the collector, the solvent evaporates and the optical fiber forms. Additionally, by redesigning the nozzle pattern and the inner structure of the syringe, electrospinning can be used to fabricate coaxial fibers from multi-composite materials [157]. Table 6 summarizes biocompatible optical fiber probes fabricated via spinning, including main materials and a brief introduction of the fabrication process, properties of obtained fibers, and their corresponding references.

### 3.3. Casting

**Figure 7:**
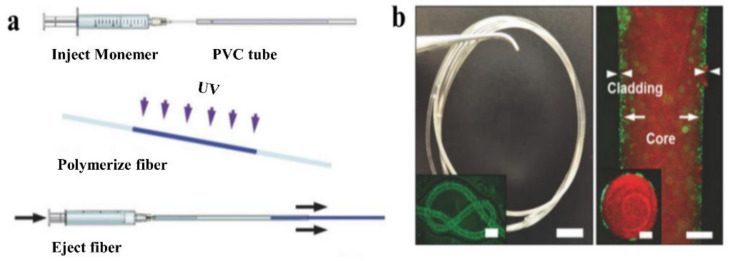
(**a**) A schematic showing the fabrication process of casting. (Reprinted with permission from ref. [46]). (**b**) Images of the obtained p(AM-co-PEGDA-co-3-APBA) based optical fiber. (Reprinted with permission from ref. [46]).

Casting, a straightforward method for fabricating preforms and optical fibers of any shape [56], is widely used because it is efficient, universal, low-cost, and simple to operate. As illustrated in Figure 7a, the process begins with the preparation of the precursor solution, continues with the injection of the solution into molds, shapes the precursor into the fiber, and removes the fiber from the mold. The products are usually isotropic due to the low cooling rate in all directions. Apart from directly fabricating preforms and fibers, casting has been used to clad an unclad fiber. For example, it was used to fabricate step-index silk waveguides with a silk fibroin core and a hydrogel cladding [68].

For instance, the first glucose-sensitive hydrogel optical fiber was fabricated via casting with a core of poly(acrylamide-co-poly(ethylene glycol) diacrylate) 3-(acrylamido) incorporated with phenylboronic acid (3-APBA) p(AM-co-PEGDA-co-3-APBA) and a Ca alginate cladding [46]. The prepared AM and PEGDA monomer solution was injected into a poly(vinyl chloride) (PVC) tube and exposed to UV light for crosslinking. The fiber core was then removed from the tube and immersed in a Na alginate solution, and then in a CaCl_2_ solution to form a Ca alginate hydrogel cladding. The entire process took approximately 5 min, making it both simple and time-efficient. Within specified limits, the thickness of the cladding increases as the concentration of Na alginate is increased.

Additionally, the recently reported optical fiber based on PEGDA for glucose monitoring was made by casting [95]. The fabrication process consists of the following four steps: (1) making the hydrogel precursor; (2) milling the asymmetry microlens array; (3) blending the 3-APBA; and (4) attaching the prepared hydrogel sensor to the fiber’s tip. The entire procedure took approximately 5 min. The simplicity and effectiveness of the fabrication process were the proposed fiber probe’s primary advantages. Table 7 summarizes optical fiber probes fabricated via casting, including main materials, a brief introduction of the fabrication process, properties of obtained fibers, and their corresponding references.

Casting has a lot of benefits, but it also has some drawbacks. To begin, despite the ease with which various shapes can be produced, complex structures within the fiber via casting are difficult to fabricate. Thus, the products are frequently intermediates in other processes, such as thermal drawing, or they may require additional processing to yield the desired fiber probe. Additionally, mass production of small-diameter functional optical fibers via casting remains a challenge.

## 4. Biomedical Applications for Biocompatible and Biodegradable Optical Fibers

The optical fiber application requirements in the biomedical field are distinct from those in other fields. Along with meeting the required light-guiding capability of optical fiber, it is critical that optical fibers cause as little damage to the invaded tissue as possible. Thus, optical fibers must meet biocompatibility and biodegradability requirements. Wang et al. conducted a review of integrated passive and active optical devices with sensing modules that are used in medical fields, as well as wearable optical devices for light therapy [40]. In Table 8, we provide an overview of typical biocompatible optical fiber, including their attenuation, typical refractive indexes, Young’s modulus, stretchability, and lifespan. The applications of biocompatible and biodegradable implantable optical fibers for deep-tissue light guiding are divided into the following categories: clinical treatment, optogenetics, biosensing, drug delivery, and neural recording.

### 4.1. Clinical Treatment

**Figure 8:**
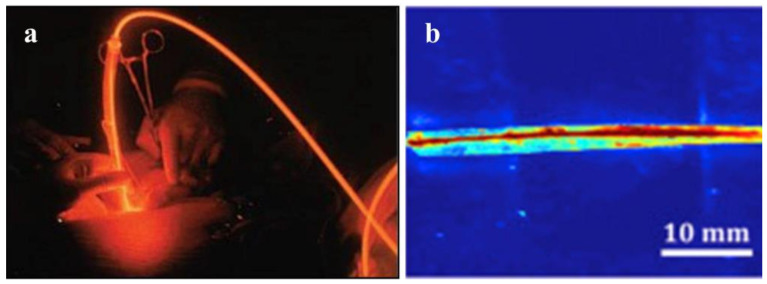
(**a**) An example of photodynamic therapy. (Reprinted with permission from ref. [55]). (**b**) Side view of light delivery using the citrate-based fiber, with the POC cladding and POMC core using a two-step fabrication method. (Reprinted with permission from ref. [67]).

Photonic applications, such as laser therapy and light activation therapy, are being used in increasing numbers of clinical treatments (Figure 8a). Lasers were first used in 1960 by Maiman, who invented the ruby laser. Since then, researchers have investigated additional laser applications in a variety of fields, including the rapidly growing field of biomedicine. With a global market value of more than USD 3 billion [169], laser therapy is widely used in ophthalmology, dermatology, urology, gastroenterology, cardiology, and cardiovascular and cerebrovascular diseases. Light-activated therapies make use of light’s inherent versatility to manipulate photoactive molecules, proteins, or cells in order to accomplish a therapeutic goal. Photodynamic therapy (PDT) is a critical component of cancer treatment because it aggregates special photosensitizers around target cells via optical absorption, thereby limiting the target cells’ oxygen and nutritional supply and killing specific tumor cells [170]. Nowadays, it is used to treat bladder, brain, esophageal, lung, ovarian, and skin cancers in clinical settings [171,172]. The global market for phototherapy and photodynamic therapy drugs and equipment was estimated to be USD 630 million in 2014 [170].

However, the clinical application of PDT is still constrained by factors such as light penetration into biological tissues. Light penetrates less than 1 cm directly, and light transmission in deep tissue requires a minimally invasive insertion technique. Furthermore, optical fiber’s biological incompatibility precludes long-term tumor treatment [173]. As a result, it is critical to develop more functional optical fibers suitable for PDT. Numerous optical fibers are being developed to deliver light deep into tissues, with some already being used in PDT and laser surgery. One type of biocompatible optical fiber is entirely composed of silk fibroin, a biodegradable and environmentally friendly material that not only has excellent stability, flexibility, and a low transmission loss rate (2 dB/cm), but is also biocompatible. The core of the waveguide was a long narrow strip of silk film (refraction index of 1.54, 2.9 mm wide), which was surrounded by a silk hydrogel (refractive index: 1.34) [68]. Qiao X, et al. have fabricated an optical fiber using genetically engineered spider silk, which possesses significantly lower attenuation of 0.8 ± 0.1 dB/cm in the air than that of regenerative silkworm silk optical fiber. The proposed recombinant spider silk optical fiber is a promising candidate for biocompatible implants with light-guiding efficiency and biodegradability [76]. Another polymeric optical fiber (core: 500 μm) based on citrate has also been reported to be capable of imaging and deep-tissue light propagation in vivo (Figure 8b). Due to the fact that this type of fiber is biodegradable and nontoxic, it can remain in a patient’s body following the treatment and degrade naturally rather than being surgically removed [67], which not only reduces the patient’s pain and medical expenses, but also improves the curative effect. Different concentrations of polymer degradation products were tested on 3T3 fibroblast cells and found to have low cytotoxicity, which was a considerable change from the FDA-approved PLGA [67]. After a subcutaneous implantation study with SD rats, there was no significant difference in in vivo foreign body responses between the POC or POMC films and the PLLA film (which is commonly utilized in the biomedical implantation field). The implanted membrane only induced a little chronic inflammatory reaction after 8 weeks. All of this research demonstrated that this fiber and its degradation products were biocompatible [67]. Additionally, due to its biocompatibility and excellent optical properties, poly (D, L-lactic acid) (PDLLA) has been suggested as a suitable material for implantable optical fiber. PDLLA optical fibers have the lowest optical propagation loss (0.11 dB/cm at 772 nm) of any bioabsorbable optical fibers [167], diameter from 2 mm down to 120 μm. Moreover, it had marvelous biodegradability. The degradation study showed that the remaining molecular weights are 16% (200 μm diameter fibers), 13% (300 μm diameter fibers), and 12% (600 μm diameter fibers) of the initial values after 100 days [167].

### 4.2. Optogenetics

**Figure 9:**
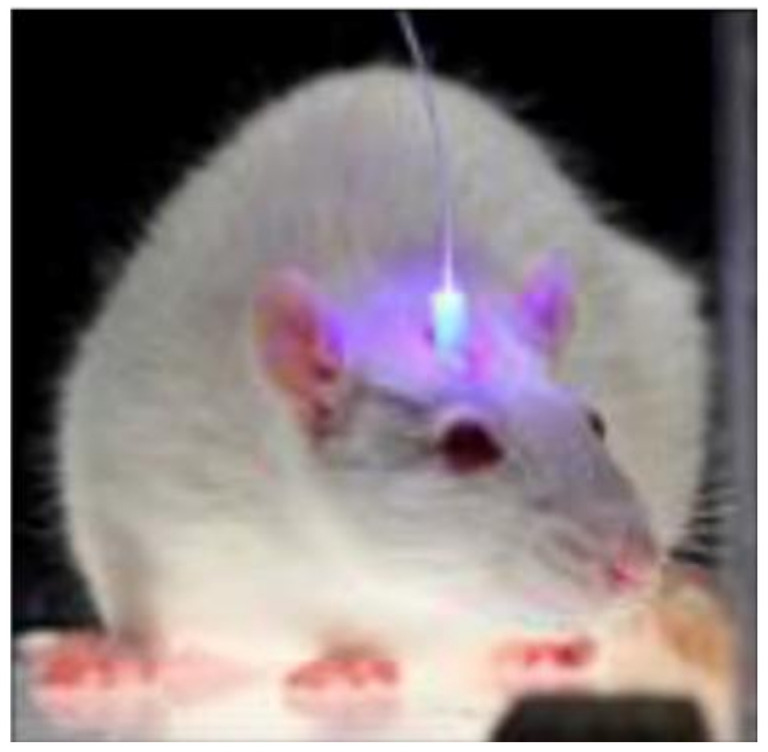
A fiber implanted into a freely behaving mouse. (Reprinted with permission from ref. [55]).

Optogenetics, a flourishing technology that combines genetics and optics, has been widely applied in a variety of fields, including behavioral science and neuroscience. A Stanford University professor published in 2005 that ChR2 (Channelrhodopsin-2), a light-sensitive protein of Chlamydomonas, was transfected into neurons via virus vector and that the neurons could be excited by external light [174]. Optogenetics technology has advanced rapidly since then, and it has been used in clinical trials on mammals over the last decade (Figure 9). Many different brain regions and neurons have had their specific roles clarified using this technology. Optogenetics technology can achieve precise control of target cells by utilizing high temporal and spatial resolution [175]. Its enormous potential has been discovered in the treatment of chronic pain [176], cardiac disease [177], epilepsy [178,179], depression [180], and Parkinson’s disease [181].

Optogenetic technology combines optical and genetic technologies to control the excited or inhibited state of neurons. Transgene technology allows photosensitive proteins, such as ChR2 (which excites neurons) and NpHR (which inhibits neurons), to be expressed specifically on the membrane of neurons. Photosensitive proteins respond to different wavelengths [182]. The neurons will be controlled by light after transfection. However, due to the tissue’s low light penetration, light conduction must be accomplished using an implantable optical fiber. As a result, optogenetic technology relies heavily on optical fiber and photosensitive proteins. Optical fibers used for optical import are typically silicon-based in traditional optogenetic applications. Despite having low light loss, high stability, and low cost, Young’s modulus of the fibers is far from that of biological tissues. This poor compatibility can easily result in tissue damage, inflammation, and immune rejection [183], which is not ideal for long-term implant research.

Numerous optogenetic implantable flexible fibers developed by researchers in recent years have improved in terms of biocompatibility and biodegradability, making significant contributions to the field of optogenetics. One type of implantable biodegradable fiber (diameter: 220 μm) based on PLLA has been shown to be an excellent tool for intracranial light transmission in order to achieve deep brain optogenetic stimulation in mice [45,165]. Although lesions are present when PLGA fibers are implanted in the mouse brain at first, lesions in the brain region rapidly diminish and almost disappear once the fibers have entirely degraded [45]. Additionally, there is a PAAm hydrogel optical fiber (diameters: 125 μm, 250 μm, and 500 μm) with a low Young’s modulus and high elasticity that is more compatible with nerve tissue and has the potential to reduce the immune responses at the nerve interface. Additionally, it has been demonstrated that it has a low light propagation loss and that optogenetic stimulation can somewhat regulate the behavior of a free-moving animal [62]. The tissue response at the silica and hydrogel optical implants were substantially lower (*p* < 0.005) in the hydrogel group than in the silica group according to quantitative analysis [62]. These studies will aid in the exploration of novel materials for optical fibers used in optogenetics that will compensate for the shortcomings of conventional silicon-based fibers. Apart from the light-transmitting fiber, numerous functional probes have been developed in recent years. In free-moving mice, a flexible micro-multimaterial fiber probe (core: 3 mm; inner clad: 8 mm; outer clad: 30 mm) exhibits remarkable transmittance, stability, and histocompatibility. Additionally, a single fiber probe can perform nerve stimulation and electrophysiological signal acquisition simultaneously, with a signal-to-noise ratio that is more than six times that of the reference probe [184].

### 4.3. Biosensing

Biosensors are devices that can detect bioactive compounds and generate quantifiable signals as a result of chemical reactions. They are typically composed of a bioreceptor, a base material, a transducer, and an electronic component [185]. Biosensing is an efficient method for studying the metabolism and activity of living cells and has significant applications in rapid diagnosis, physiology, and pathology. It is generally possible to monitor some biomarkers in the body qualitatively during disease treatment, and the resulting physiological data is critical for disease course analysis and disease judgment. To be more precise, hemoglobin is a critical marker for a variety of clinical diseases [186,187,188]. During wound healing, the pH value [189] and pressure [190] are useful indicators for determining the degree of infection and healing. Along with numerous mental diseases [191] and addiction, it has been found that the neurotransmitter dopamine (DA) interacts with tumor immunity [192].

Optical fiber detection is a common type of biosensor. Optical monitoring, which utilizes the interaction of light and biological tissues, enables the monitoring of specific substances in organisms, with the advantages of minimal tissue invasion and high signal sensitivity [55]. Optical sensing technology has several advantages over electrochemical sensing technology, including the ability to conduct real-time continuous monitoring for an extended period of time without the use of markers. Although optical sensing still has significant limitations in terms of deep-tissue sensing and real-time sensing, optical sensors have significant application potential in clinical medicine.

**Figure 10:**
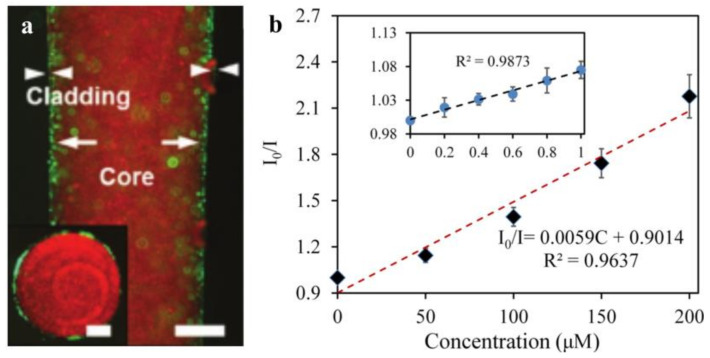
(**a**) A fluorescent image of the hydrogel fiber (scale bar = 500 µm) and the inset image is the fiber cross-section (scale bar = 250 µm). (Reprinted with permission from ref. [46]). (**b**) The calibration curve of the UCNPs-HOF fiber detecting DA in the range of 0–200 μM, and the inset shows a linear plot in a small range (0–1 μM). (Reprinted with permission from ref. [193]).

One type of hydrogel fiber (diameter: 200 μm) that enables quantitative glucose detection in vivo is composed of 3-APBA molecules functionalized with PEGDA molecules in the core and a Ca alginate cladding (Figure 10a). The complexation of the 3-APBA molecule with glucose cis-diol alters the diameter of the hydrogel fiber and, thus, responds to changes in the optical fiber’s refractive index [165]. By analyzing the optical transmission loss, the glucose content in the physiological range can be quantitatively monitored by inserting subcutaneous tissue fluid [46], which is a critical concept for glucose optical monitoring. Additionally, the optical DA sensor is beneficial for quantifying in vivo DA data acquisition. However, because of its lack of selectivity and biocompatibility, its clinical application is severely limited. Another biocompatible, soft hydrogel optical sensor is capable of detecting and quantifying DA. It is composed of hydrogel fibers and upconversion nanoparticles (UCNPs) that can detect DA molecules via the luminescence energy transfer (LET) between the UCNPs and the DA oxidation products. As illustrated in Figure 10b, it exhibits a high linear response (200 μmol/L) and a relatively low detection limit (83.6 nmol/L) [193]. Additionally, a wound healing monitoring bandage is available that measures the wound’s pH and pressure. The dye rhodamine B was doped into a precursor of PDMS, and then embedded in gauze and hydrogel wound dressings. Pressures as low as 0.1 kPa can be measured using fiber-encapsulated bandages. Additionally, it can function as a pH detector, as an increase in the pH value results in a linear decrease in the transmitted light’s power (R^2^ = 0.998) [194].

### 4.4. Drug Delivery and Neural Recording

Due to the advancement of single-function optical fibers made from a variety of materials, integrating multiple functions into a single fiber has become popular. Numerous synthetic materials exhibit desirable physical properties, such as translucence, softness, flexibility, chemical stability, and biological affinity. However, in addition to their primary function of transmitting light, an increasing number of implantable optical fibers are being developed for biomedical applications, such as drug delivery regulation [58] and electrophysiological signal recording [54]. One type of biodegradable optical fiber with multiple functions has a unique structure with a small hole in the inner core cellulose tube, integrating fluid and drug release functions. Due to the controllable collapsing or opening state, it is ideal for applications involving drug release regulation.

**Figure 11:**
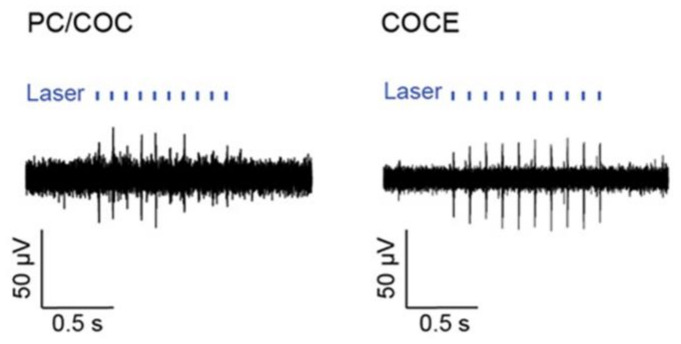
The figure shows the neural activity evoked by optical stimulation delivered through the PC/COC fiber (or COCE fiber) and recorded with the concentric AgNW mesh electrodes in a spinal cord of a Thy1-ChR2-YFP mouse. (Reprinted with permission from ref. [118]).

Optogenetics technology is critical in brain and neural science because it allows for simultaneous manipulation and detection of specific neurons. As a result, the development of neural probes that combine light transmission and cell recording functions also holds considerable promise [122,195]. The analysis of neural circuits requires not only precise stimulation of specific neurons to a millisecond resolution, but also high-resolution neural recording equipment. Currently, the elastic modulus of the most used electrophysiological probe is significantly greater than that of nerve tissue [54], resulting in the death of neurons surrounding the implanted electrode [196]. Additionally, the efficacy gradually decreases over the course of a long-term detection. To overcome these constraints, the development of noninvasive stimulation and recording probes is critical. A flexible concentric probe (core diameter: 100 to 130 μm) constructed from polymer fibers and nanowires can be used to simultaneously stimulate the spinal cord optically and record electrophysiological data in freely moving mice [118] (Figure 11). An optical fiber probe enables the integration of a variety of interrogation methods into the nerve probe via the application of the thermal stretching process. It is capable of optogenetic stimulation in free-moving mice, as well as nerve recording and drug delivery in parallel. The fibers’ long-term stability is also exceptional, allowing for at least two months of the stable brain–computer interface [54].

## 5. Conclusions

This paper examines recent developments in biocompatible and biodegradable POFs. First, different types of biocompatible fibers and their properties are discussed. Then, several common optical fiber fabrication methods are discussed in depth. Finally, some clinical applications of biocompatible and biodegradable POFs are discussed, including optogenetics, biosensing, drug delivery, and neural recording. Biocompatible fiber has a greater potential for use in biomedical applications than standard silicon-based fiber. Because of its adaptability, it can adapt to various biological interfaces and is suitable for a wide range of complex applications. Furthermore, the biocompatibility of flexible fibers can reduce the inflammatory response of contact tissues, making it easier to monitor long-term physiological signals. With the advancement of various manufacturing methods, optical fiber has a wide range of applications in implant equipment.

However, the current biocompatible optical fibers still fail to achieve large-scale commercialization. We believe that the reasons are as follows. The first is the loss of optical propagation in biocompatible fibers. The optical loss of the latest biocompatible and biodegradable optical fibers is significantly higher than that of conventional silica-based optical fibers. Second, biomedical applications should further realize the material or fiber design’s specific functionalization. Different research or clinical applications necessitate the use of different equipment to achieve the desired results. The degradation, for example, will reduce the long-term measurement efficiency of biodegradable optical fiber. However, secondary surgery can be avoided in some cases due to the degradation of optical fibers, reducing the financial burden and suffering of patients. Third, the difficulty of implantation of flexible fiber is positively correlated with its flexibility. The implant method must also be optimized. Overall, biodegradable optical fibers have a high application potential, opening up new avenues for biomedicine. From our perspective, future biocompatible optical fiber research should exploit or improve materials and fabrication methods based on the needs of optical transmission and low immune response. It develops in two directions, namely, implantable optical fibers that can achieve biodegradation or optical fibers with sufficient stability to meet long-term monitoring.

## Figures and Tables

**Table 1:** Classification of biocompatible polymer optical fiber.

Subchapter	Category	Material
2.1	Natural materials	Silk Cellulose Bacteria-cell-based
2.2	Hydrogel	PEG ^1^ PEGDA ^2^ PAAm ^3^
2.3	Synthetic	PLA ^4^, PGA ^5^, PLGA ^6^ PLLA ^7^
2.4	Elastomers	PDMS ^8^ POC-POMC ^9^
2.5	Multifunctional	PC ^10^, COC ^11^, CPE ^12^

^1^ Poly (ethylene glycol). ^2^ Poly (ethylene glycol) diacrylate. ^3^ Polyacrylamide. ^4^ Poly (lactic acid). ^5^ Poly (glycolic acid). ^6^ Poly (lactic-co-glycolic acid). ^7^ Poly (L-lactic acid). ^8^ olydimethylsiloxane. ^9^ Poly (octamethylene citrate)-poly (octamethylene maleate citrate). ^10^ Polycarbonate. ^11^ Cyclic olefin copolymer. ^12^ Conductive polyethylene.

**Table 2:** Main characteristics of the hydrogel materials.

Name	Characteristics	Ref.
PEG	Colorless viscous liquid Soluble in water, ethanol, benzene, and acetone Inert, odorless, and nonvolatile nD/20: 1431–1433 Weak toxic and nonimmunogenic	[96]
PEGDA	Hydrophilic and elastic hydrogel Melt point: 12–17 °C n20/D: 1.47 Nontoxic	[97]
AAm	White powder Odorless Soluble in water and alcohol	[98]

**Table 3:** Main characteristics of the synthetic polymer materials.

Name	Characteristics	Ref.
PLA	Density: 1.25–1.28 g/mL Flexural modulus: 100–150 MPa Tensile strength: 40–60 MPa Nontoxic	[99]
PGA	Crystallinity degree: 45–55% Melting temperature: 220–230 °C Mechanical strength: 115 MPa Weak toxic	[100]
PLGA	Crystallinity degree: 45–55% Melting temperature: 220–230 °C Mechanical strength: 115 MPa Weak toxic	[100,101]
PLLA	Flexural strength: 48–110 MPa Tensile strength: 61–66 MPa Weak toxic	[102]

**Table 4:** Main characteristics of the elastomer materials.

Name	Characteristics	Ref.
PDMS	Colorless Volatile liquid High viscosity Nontoxic	[105]
Citric acid	Density: 1.542 g/cm^3^ Soluble in water Decompose at 175 °C Nontoxic	[106]
1,8-octanediol	Melting point: 57 °C–61 °C Soluble in water and methanol.	[107]
Maleic anhydrate	Melting point:51 °C–54 °C Low toxicity and health hazard Boiling point: 202 °C	[108]

**Table 5:** Preparation methods of preform constituents of multifunctional fiber.

Metal	Semiconductor	Polymer
Milling Powder Consolidation Casting	Evaporation Powder Consolidation Milling	Casting Milling Rolling Laser-cutting

**Table 6:** Summary of biocompatible optical fiber probes fabricated via spinning, including main materials, brief introduction of the fabrication process, properties of obtained fibers, and their corresponding references.

Materials	Fabrication Methods	Fabrication Process	Properties of Fibers	Ref.
PEGDA, AAm, Na-alginate, CaCl_2_	Dynamic wet spinning	Preparing the core and sheath spinning solutions and extruding into a coagulation bath, illuminating by 360 nm UV light	Optical attenuation of 0.18 ± 0.01 dB/cm at 650 nm; Young’s modulus < 2.6 MPa	[88]
Silk, jute fibers	Microfluidic wet spinning	Preparing RSF solution, CNF suspension, and RSF/CNF suspensions, extruding the precursor to a bath containing 95 vol% ethanol aqueous solution	Optical attenuation of 0.1 dB/cm; breaking strength of 710.2 ± 33.2 MPa	[158]

**Table 7:** Summary of optical fiber probes fabricated via casting, including main materials, brief introduction of the fabrication process, properties of obtained fibers, and their corresponding references.

Materials	Fabrication Process	Properties of Fibers	Ref.
PLA and PLGA	Melting the powders, pressing and annealing the melts into films, and laser cutting films into fibers	Planar waveguide; attenuation of 1.6 dB/cm at 635 nm	[65]
PEGDA, AM and 3-APBA	Casting the precursor solution into a mold to form the core and immersing the core in Na alginate and CaCl_2_ solution to form the cladding	Glucose-sensitive probe; attenuation of 0.2 dB/cm at 400 nm (p(AM-co-PEGDA) mol % = 90%)	[46]
PEGDA, AM, 3-APBA and N, N′-methylenebisacrylamide	Preparing the precursor, machining the asymmetry microlens array, blending the 3-(acrylamido)phenylboronic acid, and attaching the prepared hydrogel sensor to the fiber’s tip	Glucose-sensitive probe; 0.4 dB/cm at 532 nm (PEGDA precursor concentration of 90 vol %); sensitivity of 2.6 μW/mM	[95]
Silk fibroin and silk hydrogel	Casting the silk solution (silk film) into a mold and dip-coating the core in silk hydrogel (silk hydrogel *n* = 1.34) solution	Step-index optical finer; optical attenuation of about 2 dB/cm at 540 nm	[68]
Spider silk protein and silkworm silk protein	Dissolving the spider silk protein in hexafluoro-2-propanol at 37 °C for a night and casting the silkworm silk solution in a tube, heating the molds filled with protein solutions at 60 °C for seven days	Biodegradable optical fiber; optical attenuation of 0.8 dB/cm st 635 nm	[76]
PEG and sodium alginate	Preparing the PEG precursor and injecting it into a tube, illuminating the tube by UV light, coating the core by dipping it in a sodium alginate and calcium chloride	Step-index waveguide; optical attenuation of 0.42 dB/cm at 492 nm	[85]
PAAm hydrogel and Ca^2+^ with Na alginate	Preparing the acrylamide with Na alginate precursor, injecting the solution to a tube mold, and illuminating the tube by UV at 50 °C for 30 min	Unclad strain sensing optical fiber; optical attenuation of 0.56 dB/cm at 532 nm	[90]
Agarose	Boiling the agar and pouring it into a glass mold,	Core-cladding fiber probe; optical attenuation of 3.32 dB/cm at 633 nm	[159]

**Table 8:** Several typical polymeric biocompatible optical fibers and their properties.

Optical Fiber	Attenuation (dB/cm)	Refractive Index	Young’s Modulus	Stretchability (%)	Lifespan	Ref.
Silkworm silk	0.22 (632.8 nm)	1.54–1.55	5–12 GPa	4–16	n.r. ^a^	[69,112]
Spider silk	0.7–10.5 (635 nm)	1.5–1.7	1–24 GPa	4–33	2–3 weeks	[76,160]
Cellulose	1–2 (630 nm)	1.475	n.r. ^a^	30–70	≥90 days	[58,161]
PEG hydrogel	1–6 (532 nm)	1.35–1.47	1–44 kPa	300–2000	n.r. ^a^	[162]
PDMS	1.8 (532 nm)	1.41–1.47	0.6–2.6 MPa	95–140	n.r. ^a^	[163,164]
PLLA	1.5–1.6 (473 nm)	1.46–1.47	2.7–7 GPa	3–100	12–18 weeks	[165,166]
PDLLA	0.11 (772 nm)	n.r. ^a^	n.r. ^a^	3–10	11–15 weeks	[167]
PLGA (50/50)	n.r. ^a^	1.47–1.6	0.7–7 GPa	7–20	1–25 weeks	[165]
POC and POMC	0.4–2 (633 nm)	1.5–1.54	4.7–6 MPa	50–100	4–6 weeks	[67,168]

^a^ not reported.

## Data Availability

Not applicable.
